# Estimation of Socioeconomic Inequalities in Mortality in Japan Using National Census-linked Longitudinal Mortality Data

**DOI:** 10.2188/jea.JE20210106

**Published:** 2023-05-05

**Authors:** Hirokazu Tanaka, Johan P. Mackenbach, Yasuki Kobayashi

**Affiliations:** 1 Department of Public Health, Erasmus University Medical Center, Rotterdam, The Netherlands; 2 Department of Public Health and Occupational Medicine, Graduate School of Medicine, Mie University, Mie, Japan; 3 Department of Public Health, Graduate School of Medicine, The University of Tokyo, Tokyo, Japan

**Keywords:** deterministic linkage, mortality inequalities, socioeconomic inequalities, census, vital statistics

## Abstract

**Background:**

We aimed to develop census-linked longitudinal mortality data for Japan and assess their validity as a new resource for estimating socioeconomic inequalities in health.

**Methods:**

Using deterministic linkage, we identified, from national censuses for 2000 and 2010 and national death records, persons and deceased persons who had unique personal identifiers (generated using sex, birth year/month, address, and marital status). For the period 2010–2015, 1,537,337 Japanese men and women aged 30–79 years (1.9% in national census) were extracted to represent the sample population. This population was weighted to adjust for confounding factors. We estimated age-standardized mortality rates (ASMRs) by education level and occupational class. The slope index of inequality (SII) and relative index inequality (RII) by educational level were calculated as inequality measures.

**Results:**

The reweighted sample population’s mortality rates were somewhat higher than those of the complete registry, especially in younger age-groups and for external causes. All-cause ASMRs (per 100,000 person-years) for individuals aged 40–79 years with high, middle, and low education levels were 1,078 (95% confidence interval [CI], 1,051–1,105), 1,299 (95% CI, 1,279–1,320), and 1,670 (95% CI, 1,634–1,707) for men, and 561 (95% CI, 536–587), 601 (95% CI, 589–613), and 777 (95% CI, 745–808) for women, respectively, during 2010–2015. SII and RII by educational level increased among both sexes between 2000–2005 and 2010–2015, which indicates that mortality inequalities increased.

**Conclusion:**

The developed census-linked longitudinal mortality data provide new estimates of socioeconomic inequalities in Japan that can be triangulated with estimates obtained with other methods.

## INTRODUCTION

Monitoring socioeconomic inequalities in health represents an initial step towards achieving equity in society.^1^^,^^2^ Socioeconomic inequalities in mortality have been assessed in most high-income countries, including European countries,^3^^–^^7^ the United States,^8^ Canada,^9^ Australia,^10^ New Zealand,^11^ and Korea.^12^ These studies, especially those examining education-based inequalities,^13^ were generally conducted using national-census-linked longitudinal mortality data that covered entire populations or nationally representative populations. However, socioeconomic inequalities in mortality in Japan remain relatively understudied; this is because there is no national longitudinal mortality database that also features data regarding socioeconomic status.^14^

Although studies have examined mortality inequalities using data from the Japanese national register, finding that the inequalities between Japan’s occupational classes are smaller than those in European countries,^15^^–^^17^ these studies generally applied cross-sectional approaches, which risk numerator-denominator bias.^15^^–^^17^ Further, no cross-sectional mortality data suitable for determining mortality inequalities by education level are available for the Japanese population because educational background is not surveyed in the national death registry. A recent study used national census and death records to estimate changes in Japan’s mortality inequalities by education level^18^; however, estimating mortality rates by socioeconomic status remains limited by the available data: this previous study used only educational attainment as a socioeconomic status indicator.^18^ Moreover, the study allowed 1:n matching, which distributes one death count (numerator) to N matched census cases (denominator) depending on the percentage of educational attainment averaged out by a key matching variable. This would cause systematic underestimations of mortality inequalities even if there were inequalities by educational attainment. Here, use of 1:1 matching linkage allows us to overcome this limitation.

Furthermore, there is currently no suitable national database for mortality-related socioeconomic inequalities in Japan. This especially obstructs attempts to address national mortality inequalities in accordance with measures for addressing global health inequalities. Individual linkage between census and death-record data might resolve this issue. Thus, this study aimed to develop census-linked longitudinal mortality data and assess its validity as a new resource. This included estimating mortality rates by socioeconomic status for the Japanese population, which would enable international comparisons. Such research could contribute useful benchmarks and entry points for monitoring and reducing socioeconomic inequalities in health.

## METHODS

### Data sources

We used data from the Population Census (hereafter, ‘the census’), conducted quinquennially by the Ministry of Internal Affairs and Communications (MIC),^19^ and the National Vital Statistics (hereafter, ‘death records’), collected annually by the Ministry of Health, Labour and Welfare (MHLW).^20^ Anonymized microdata were extracted and used with permission from the MIC and MHLW.

We extracted from the censuses conducted on October 1, 2000, and October 1, 2010, individual data for all Japanese nationals living in Japan (denominator: person-years at risk). Regarding death records (numerator), two periods were examined: October 2000–September 2005 (wave 1), and October 2010–September 2015 (wave 2). Foreigners living in Japan were excluded.

### Deterministic linkage and personal identifiers

We applied a deterministic-linkage method using ‘personal identifiers’ (IDs). We generated these IDs because there is no official personal identification code (eg, national security number) for linking national statistics and survey data in Japan. Each ID comprised five variables: sex, birth year, birth month, address (municipality-level local government code), and marital status (single, married, widow, divorced, or unknown). Day of birth was not surveyed in the census; exact address (eg, postcode, house number) was not available because of privacy protection. The deterministic linkage and all analyses described below were conducted for wave 1 and wave 2. eTable 1 shows the distribution of the population from the 2010 census and the deceased persons for wave 2 in terms of numbers of people with unique IDs and duplicated IDs, respectively.

**Figure 1.**
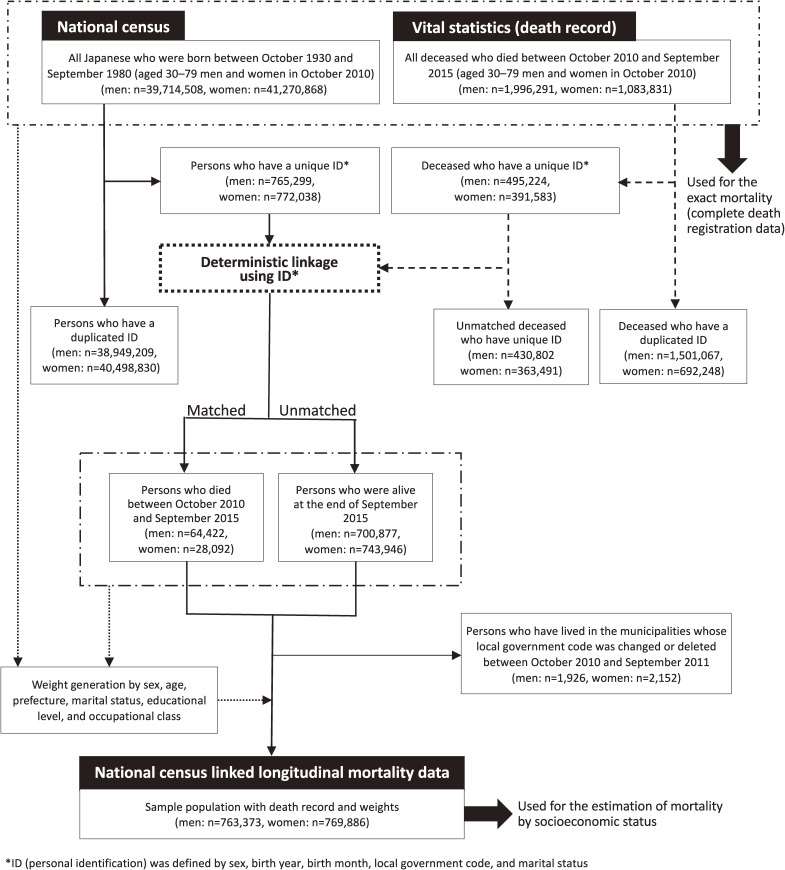
Deterministic linkage procedure for linking the population census and vital statistics data for 2010–2015.

Figure 1 shows the deterministic-linkage procedures for wave 2. First, the population in 2010 and all deaths in wave 2 were counted, which indicated the exact mortality (hereafter, ‘complete registry’). Second, we identified persons from the census and death records who had unique IDs, respectively; 1.9% of the population from the 2010 census and 886,807 deceased persons (from wave 2) were identified as having unique IDs, respectively.

Third, deterministic linkage was conducted using the individuals with unique IDs. If a person from the census was not matched to a deceased person with a unique ID, we considered him/her to have been alive at the end of the follow-up period. For wave 2, 64,422 men and 28,092 women were matched with persons from the census, meaning 700,877 men and 743,946 women were presumed alive at the end of September 2015. Fourth, we excluded persons who had lived in municipalities for which the local government code was deleted during the first year of the follow-up period; this was to ensure at least 1 year of follow-up. Finally, we developed census-linked longitudinal mortality data that included demographics, socioeconomic status, year and month of death, cause of death, and date of censoring due to local-government code change. Underlying causes of death were classified according to the International Statistical Classification of Diseases, 10^th^ Revision, and grouped into four broad groups: cancer (C00–D48) cardiovascular diseases (I00–I99), external causes (V01–Y98), and all other diseases, respectively.

### Weighting

We sampled persons who had unique IDs from all Japanese people aged 30–79 years. This method assumes that, if all variables are evenly distributed across individuals, random sampling will occur. However, birth year (age), municipalities, and marital status are unevenly distributed in the Japanese population.^19^ Therefore, we weighted the sampled population to adjust for confounding between the distribution of demographics and mortality.^21^

In our procedure, persons who lived in municipalities with large populations were less likely to be sampled because ID duplication was more likely. Similarly, married persons were less likely to be sampled because most (approximately 70%) Japanese people aged 30–79 years were married in 2010. Therefore, when calculating mortality, married persons and people living in large municipalities should be allocated larger weights. We calculated the weighting score using ratios representing the number of population members that possessed a certain weighting key divided by the number of persons in the sample with a matching key. The weighting key (maximum: 110,920 combinations) was based on prefecture, sex, 5-year age category, marital status, education level, and occupational class (for people aged 30–64 years only). For example, suppose that 10 single men aged 30–34 years who were manual workers, lived in Tokyo, and had low education levels were observed in the census, and five men with the same demographics were observed in the sample population; a weighting score of ‘2’ (= 10/5) would be allocated to each sampled person. The range of the weighting score was set to 1–10,000 to avoid overweighting individuals; all weighting above 10,001 was set to ‘10,000’. eTable 2 shows the weighting-score calculations. We generated and allocated 71,991 weighting scores for the sample population from wave 2. Lastly, the weighting scores were recalibrated to ensure that the average weight for all sample populations was equal to one, which resulted in the standard errors being approximated to those of the unweighted sample when calculating mortality.

### Socioeconomic status

We assessed socioeconomic status using education level and occupational class.^22^ Education level was classified into three categories: ‘low’ (defined as 1–2 on the International Standard Classification of Education^23^), ‘middle’ (3–4), and ‘high’ (5–8). Occupational class was classified into five categories (based on the Erikson-Goldthorpe-Portocarero scheme^24^): upper non-manual worker, lower non-manual worker, manual worker, farmer, and self-employed. Those labelled ‘unemployed’ in the census were coded as ‘unemployed’. Detailed classifications are presented in eTable 3 and eTable 4.

### Mortality calculations

All analyses were conducted based on sex, 5-year category, and marital status. First, age-standardized mortality rates (ASMRs) among the entire population were calculated (ie, complete registry), followed by ASMRs among the unweighted sample population and ASMRs among the reweighted sample population. The 2013 European Standard Population was used as a reference for direct standardization, because the distribution is similar to that observed in the 2000 Japanese Census.^22^^,^^25^ Persons who lived in municipalities for which the local-government code was deleted between October 2011 and September 2015 were censored at the end of the prior September. To assess validity, we compared the mortality rates of the reweighted sample population with those for the complete registry.

After considering the accuracy of the unique ID and checking validity, we excluded men and women aged 30–39 years from the estimations of mortality by socioeconomic status because of overestimations among younger age-groups. Finally, we estimated ASMRs by educational level (40–79 years) and occupational class (40–64 years) for each period using the reweighted sample population.

### Inequality measures

Mortality rate difference (RD) and mortality rate ratio (RR) of low versus high educational level and manual versus upper non-manual workers were calculated to measure inequalities. We used a bootstrap procedure with 1,000 replications to calculate 95% confidence intervals (CIs). The slope index of inequality (SII) and its relative counterpart, the relative index of inequality (RII), were calculated as inequality measures for educational level.^26^ Both SII and RII were adjusted by 5-year age groups. The average inter-group differences (AID) were calculated as inequality measures for occupational class because occupational class cannot be defined as hierarchically ordered.^15^^,^^27^

## RESULTS

### Sample population size

The sample population was 2,553,447 (3.3% of the total population: 1,240,619 men, 1,312,828 women) in wave 1 and 1,537,337 (1.9% of the total population: 765,299 men, 772,038 women) in wave 2. The results for wave 2 were generally similar to those for wave 1. From this point forward, we mainly present the results for wave 2. Results for wave 1 are shown in, eFigure 1, eFigure 2, eTable 5, eTable 6, eTable 7, and eTable 8.

### All-cause mortality for men

Table 1 shows the distribution of populations and ASMRs for men. The reweighted sample population and complete registry showed similar distributions of demographic characteristics (eg, married men – complete registry: 76.4%, reweighted sample population: 74.1%; men with high education level – complete registry: 29.8%, reweighted sample population: 30.0%). Differences in all-cause ASMRs ranged from −0.7% (75–79 years) to 82.1% (35–39 years) across the 5-year age groups. For single and married men, the ASMRs of the reweighted sample population were 9.6% lower and 9.6% higher than those of the complete registry, respectively. For men aged 40–79 years, all-cause ASMR (per 100,000 person-years) was 1,289 (95% CI, 1,287–1,290) for the complete registry and 1,373 (95% CI, 1,359–1,386) for the reweighted sample population. Among men aged 40–79 years, the reweighted sample population’s ASMRs were 6.5% higher (84 per 100,000 person-years higher) than those of the complete registry.

**Table 1.** Distribution of population and all-cause age-standardized mortality rate (per 100,000 person-years)^a^ for men during 2010–2015

	All population (complete registry)			Sample population					Comparisons with complete registry
				Unweighted			Weighted		
	Population	(%)	(A) ASMR	Number of sample	(%)	ASMR	(%)	(B) ASMR	% difference [(B) − (A)]/(A) (%)
Total (30–79 years)	39,714,508		—	763,373		—		—	
Total (40–79 years)	30,764,583		1,289	641,173		1,750		1,373	6.5
Age, years									
30–34	4,104,431	10.3	79	60,059	7.9	196	10.9	97	22.7
35–39	4,845,494	12.2	114	62,141	8.1	276	12.5	207	82.1
40–44	4,312,115	10.9	178	67,419	8.8	374	11.3	210	18.0
45–49	3,954,073	10.0	282	72,331	9.5	527	10.4	290	2.8
50–54	3,752,271	9.4	453	78,086	10.2	849	9.4	542	19.7
55–59	4,234,130	10.7	733	83,359	10.9	1,198	10.2	902	23.0
60–64	4,870,376	12.3	1,140	84,306	11.0	1,851	10.4	1,247	9.3
65–69	3,882,977	9.8	1,785	88,041	11.5	2,626	10.1	1,804	1.1
70–74	3,195,800	8.0	2,818	86,517	11.3	3,639	8.4	3,102	10.1
75–79	2,562,841	6.5	4,988	81,114	10.6	5,061	6.6	4,954	−0.7
Marital status (40–79 years)									
Single	4,235,723	13.8	2,353	157,366	24.5	2,052	15.0	2,127	−9.6
Married	23,509,251	76.4	1,049	59,267	9.2	1,097	74.1	1,150	9.6
Widow	949,355	3.1	2,149	156,645	24.4	1,744	3.4	1,754	−18.4
Divorced	1,618,652	5.3	3,271	184,461	28.8	2,470	5.8	2,696	−17.6
Unknown	451,602	1.5	68	83,434	13.0	47	1.7	35	−48.2
Educational level (40–79 years)^b^									
High (ISCED: 5–8)	9,159,211	29.8	—	88,594	13.8	1,776	30.0	1,078	
Middle (ISCED: 3, 4)	12,600,741	41.0	—	259,269	40.4	1,876	43.6	1,299	
Low (ISCED: 1, 2)	5,267,134	17.1	—	189,510	29.6	2,032	17.3	1,670	
Unknown	3,737,497	12.1	—	103,800	16.2	946	9.1	1,682	
Occupational class (EGP scheme, 40–64 years)^c^									
Upper non-manual workers (I+II)	2,749,618	13.8	—	24,483	6.8	759	13.7	303	
Lower non-manual workers (III)	5,342,019	26.9	—	54,616	15.1	790	27.7	472	
Manual workers (V+VI+VIIa)	5,619,131	28.3	—	107,061	29.6	845	29.7	507	
Farmers (IVc+VIIb)	589,377	3.0	—	27,005	7.5	808	2.9	579	
Self-employed (IVa+b)	1,809,355	9.1	—	28,709	7.9	969	8.4	448	
Unemployment	3,760,432	18.9	—	119,703	33.1	1,146	17.6	1,389	

ASMR, age-standardized mortality rate.

^a^ASMR (per 100,000 person-years) was calculated from death between October 2010 and September 2015 counted by the vital statistics. Age-standardized mortality rates were computed using the 2013 European standard population and data in 5-year age intervals.

^b^Defined by the International Standard Classification of Education (ISCED).

^c^Defined by the Erikson-Goldthorpe-Portocarero (EGP) scheme.

### All-cause mortality for women

Table 2 shows the distribution of populations and ASMRs for women. The reweighted sample population and complete registry showed comparable distributions of demographic characteristics (eg, married women – complete registry: 71.0%, reweighted sample population: 68.2%; women with high education level – complete registry: 23.5%, reweighted sample population: 23.0%). Differences in all-cause ASMRs ranged from −19.0% (40–44 years) to 12.8% (65–69 years) across 5-year age groups. For single and married women, the ASMRs of the reweighted sample population were 5.4% higher and 1.3% lower than those of the complete registry, respectively. For women aged 40–79 years, all-cause ASMR (per 100,000 person-years) was 595 (95% CI, 594–596) for the complete registry and 633 (95% CI, 624–641) for the reweighted sample population. Among women aged 40–79 years, the ASMRs of the reweighted sample population were 6.2% higher (37 per 100,000 person-years higher) than those of the complete registry.

**Table 2.** Distribution of population and all-cause age-standardized mortality rate (per 100,000 person-years)^a^ for women during 2010–2015

	All population (complete registry)			Sample population					Comparisons with complete registry
				Unweighted			Weighted		
	Population	(%)	(A) ASMR	Number of sample	(%)	ASMR	(%)	(B) ASMR	% difference [(B) − (A)]/(A) (%)
Total (30–79 years)	41,270,868		—	769,886		—		—	—
Total (40–79 years)	32,575,276		595	637,817		776		632	6.2
Age, years									
30–34	3,985,129	9.7	45	64,108	8.3	83	10.2	40	−12.0
35–39	4,710,463	11.4	67	67,961	8.8	105	11.6	61	−9.0
40–44	4,224,928	10.2	101	74,973	9.7	163	10.2	82	−19.0
45–49	3,913,112	9.5	155	82,103	10.7	252	9.4	170	9.6
50–54	3,767,198	9.1	226	85,566	11.1	375	9.0	233	2.9
55–59	4,318,109	10.5	326	84,159	10.9	517	9.5	349	7.0
60–64	5,065,610	12.3	474	78,715	10.2	746	11.1	514	8.3
65–69	4,246,615	10.3	744	78,592	10.2	1,060	10.7	839	12.8
70–74	3,705,510	9.0	1,275	77,117	10.0	1,586	9.6	1,332	4.5
75–79	3,334,194	8.1	2,437	76,592	9.9	2,533	8.6	2,574	5.6
Marital status (40–79 years)									
Single	2,532,508	7.8	1,053	172,081	27.0	1,091	8.5	1,110	5.4
Married	23,125,462	71.0	452	59,207	9.3	468	68.2	446	−1.3
Widow	4,033,679	12.4	848	151,271	23.7	835	13.5	899	6.0
Divorced	2,451,604	7.5	1,048	168,051	26.3	960	8.3	1,073	2.3
Unknown	432,023	1.3	23	87,207	13.7	18	1.5	9	−58.4
Educational level (40–79 years)^b^									
High (ISCED: 5–8)	7,642,338	23.5	—	99,665	15.6	791	23.0	561	
Middle (ISCED: 3, 4)	15,212,524	46.7	—	277,582	43.5	787	48.8	601	
Low (ISCED: 1, 2)	5,994,980	18.4	—	171,608	26.9	952	19.1	777	
Unknown	3,725,434	11.4	—	88,962	13.9	460	9.1	699	
Occupational class (EGP scheme, 40–64 years)^c^									
Upper non-manual workers (I+II)	1,895,656	9.1	—	34,558	8.7	310	9.2	257	
Lower non-manual workers (III)	7,035,794	33.9	—	129,839	32.7	314	35.7	211	
Manual workers (V+VI+VIIa)	2,473,340	11.9	—	55,179	13.9	305	11.7	168	
Farmers (IVc+VIIb)	380,700	1.8	—	10,862	2.7	248	1.5	140	
Self-employed (IVa+b)	598,061	2.9	—	18,671	4.7	408	2.2	277	
Unemployment	8,369,888	40.3	—	147,656	37.2	536	39.7	346	

ASMR, age-standardized mortality rate.

^a^ASMR (per 100,000 person-years) was calculated from death between October 2010 and September 2015 counted by the vital statistics. Age-standardized mortality rates were computed using the 2013 European standard population and data in 5-year age intervals.

^b^Defined by the International Standard Classification of Education (ISCED).

^c^Defined by the Erikson-Goldthorpe-Portocarero (EGP) scheme.

### Cause-specific mortality

Table 3 shows a comparison between the complete registry and sample population regarding broad cause-specific mortality among men and women aged 40–79 years. Differences in ASMRs between the complete registry and reweighted sample population were based on broad cause of death and sex. For men, the ASMRs (per 100,000 person-years) of the reweighted sample population were 45, 12, 11, and 14 higher than those of the complete registry for cancer, cardiovascular disease, external causes, and others, respectively. For women, the ASMRs (per 100,000 person-years) of the reweighted sample population were 2, 15, 6, and 11 higher than those of the complete registry for cancer, cardiovascular disease, external causes, and others, respectively. In percentage terms, for both men and women mortality from external causes showed the largest differences when compared with the complete registry.

**Table 3.** Comparison of all-cause broad cause-specific age-standardized mortality rate (per 100,000 person-years) by population during 2010–2015^*^

	(A) All population (complete registry)	(B) Weighted sample population	Comparisons with complete registry	
			Absolute difference	% difference [(B) − (A)]/(A) (%)
Men (40–79 years)				
All-cause	1,289	1,373	84	6.5
Broad cause death				
Cancer	522	566	45	8.5
Cardiovascular disease	307	319	12	3.8
External causes	95	106	11	11.2
Others	364	379	14	3.9
Women (40–79 years)				
All-cause	595	632	37	6.2
Broad cause death				
Cancer	253	255	2	0.8
Cardiovascular disease	142	157	15	10.7
External causes	42	48	6	14.8
Others	158	169	11	7.0

^*^Age-standardized mortality rates were computed using the 2013 European standard population and data in 5-year age intervals.

### Mortality rates by socioeconomic status

Table 4 shows, for both sexes, the estimated all-cause and broad cause-specific ASMRs by socioeconomic status. All-cause ASMRs (per 100,000 person-years, 40–79 years) for individuals with high, middle, and low levels of education were 1,078 (95% CI, 1,051–1,105), 1,299 (95% CI, 1,279–1,320), and 1,670 (95% CI, 1,634–1,707) for men, and 561 (95% CI, 536–587), 601 (95% CI, 589–613), and 777 (95% CI, 745–808) for women, respectively. For men, all-cause ASMRs (per 100,000 person-years) for upper non-manual workers, lower non-manual workers, manual workers, farmers, and self-employed (40–64 years) were 303 (95% CI, 280–326), 472 (95% CI, 451–493), 507 (95% CI, 487–526), 579 (95% CI, 514–645), and 448 (95% CI, 416–481). For women, all-cause ASMRs (per 100,000 person-years) for upper non-manual workers, lower non-manual workers, manual workers, farmers, and self-employed (40–64 years) were 257 (95% CI, 223–291), 211 (95% CI, 199–222), 168 (95% CI, 151–184), 140 (95% CI, 99–181), and 277 (95% CI, 228–325), respectively. Estimated crude mortality rates by age group and educational level are shown in eFigure 3. The mortality differences persisted across all age groups for both sexes, but exceptions were observed (eg, women aged 65–69 in wave 2).

**Table 4.** All-cause and broad cause-specific age-standardized mortality rate (per 100,000 person-years) by socioeconomic status during 2010–2015^a^

	All-cause	95% CI	Cancer	95% CI	Cardiovascular disease	95% CI	External causes	95% CI	Others	95% CI
Men										
Total (40–79 years)	1,373	(1,359–1,386)	566	(558–575)	319	(313–326)	106	(102–109)	379	(372–386)
Total (40–64 years)	616	(605–627)	223	(217–230)	160	(155–166)	82	(78–86)	148	(143–154)
Educational level (40–79 years)^b^										
High (ISCED: 5–8)	1,078	(1,051–1,105)	494	(475–513)	231	(219–243)	94	(86–101)	257	(243–270)
Middle (ISCED: 3, 4)	1,299	(1,279–1,320)	540	(527–553)	319	(309–330)	97	(92–103)	342	(331–352)
Low (ISCED: 1, 2)	1,670	(1,634–1,707)	620	(599–641)	392	(374–410)	135	(122–149)	515	(495–535)
Unknown	1,682	(1,633–1,730)	719	(688–750)	337	(315–359)	150	(135–166)	466	(440–492)
Occupational class ​ (EGP scheme, 40–64 years)^c^										
Upper non-manual workers (I+II)	303	(280–326)	174	(157–191)	38	(30–46)	44	(36–53)	45	(36–53)
Lower non-manual workers (III)	472	(451–493)	160	(148–173)	126	(115–136)	60	(53–67)	125	(114–136)
Manual workers (V+VI+VIIa)	507	(487–526)	188	(176–200)	175	(163–186)	73	(66–80)	70	(62–77)
Farmers (IVc+VIIb)	579	(514–645)	251	(211–290)	124	(93–155)	95	(65–124)	85	(59–111)
Self-employed (IVa+b)	448	(416–481)	190	(169–210)	103	(87–119)	64	(52–77)	89	(74–104)
Unemployment	1,389	(1,345–1,433)	382	(360–403)	343	(322–365)	185	(168–202)	479	(452–505)
Women										
Total (40–79 years)	632	(623–640)	255	(250–261)	157	(153–162)	48	(46–50)	169	(165–173)
Total (40–64 years)	261	(254–268)	130	(125–135)	58	(55–62)	30	(28–33)	41	(38–44)
Educational level (40–79 years)^b^										
High (ISCED: 5–8)	561	(536–587)	301	(283–319)	72	(62–81)	28	(23–32)	156	(141–170)
Middle (ISCED: 3, 4)	601	(589–613)	233	(225–240)	168	(162–175)	50	(47–54)	149	(143–155)
Low (ISCED: 1, 2)	777	(745–808)	267	(249–284)	209	(192–227)	79	(67–92)	213	(200–227)
Unknown	699	(670–728)	304	(284–324)	143	(130–155)	30	(24–37)	219	(203–234)
Occupational class ​ (EGP scheme, 40–64 years)^c^										
Upper non-manual workers (I+II)	257	(223–291)	113	(91–135)	46	(31–61)	63	(47–79)	36	(22–49)
Lower non-manual workers (III)	211	(199–222)	119	(110–128)	41	(36–46)	21	(17–24)	30	(25–34)
Manual workers (V+VI+VIIa)	168	(151–184)	88	(76–100)	29	(22–36)	26	(19–32)	21	(15–27)
Farmers (IVc+VIIb)	140	(99–181)	95	(60–130)	14	(0–27)	6	(0–13)	14	(4–24)
Self-employed (IVa+b)	277	(228–325)	126	(93–159)	48	(29–68)	37	(19–56)	57	(35–79)
Unemployment	346	(332–359)	158	(149–167)	84	(77–90)	41	(36–46)	61	(55–67)

ASMR, age-standardized mortality rate.

^a^ASMR (per 100,000 person-years) was calculated from death between October 2010 and September 2015 counted by the vital statistics. Age-standardized mortality rates were computed using the 2013 European standard population and data in 5-year age intervals.

^b^Defined by the International Standard Classification of Education (ISCED).

^c^Defined by the Erikson-Goldthorpe-Portocarero (EGP) scheme.

95% CI: 95% Confidence Interval.

### Changes in inequality measures

Table 5 shows changes in inequality measures of all-cause ASMRs by educational level and occupational class between waves 1 and 2. During the early 2000s (wave 1), the RRs between low and high education levels for those aged 40–79 years were 1.23 (95% CI, 0.91–1.55) for men and 1.09 (95% CI, 0.71–1.46) for women. During the early 2010s (wave 2), the RRs between low and high education levels for those aged 40–79 years were 1.55 (95% CI, 1.14–1.96) for men and 1.38 (95% CI, 0.99–1.77) for women. Both SII and RII by educational level increased among both sexes between waves 1 and 2: SII (per 100,000 person-years) increased from 404 (95% CI, 356–453) in wave 1 to 721 (95% CI, 668–774) in wave 2 for men, and RII increased from 1.35 (95% CI, 1.30–1.39) in wave 1 to 1.75 (95% CI, 1.68–1.82) in wave 2. For women, SII (per 100,000 person-years) increased from 85 (95% CI, 52–119) in wave 1 to 194 (95% CI, 157–232) in wave 2, and RII increased from 1.12 (95% CI, 1.08–1.18) in wave 1 to 1.31 (95% CI, 1.23–1.39) in wave 2.

**Table 5.** Changes in inequality measures of all-cause age-standardized mortality rate by educational level and occupational class

	2000–2005 (wave 1)		2010–2015 (wave 2)	
	Point estimates	95% CI	Point estimates	95% CI
Men				
Educational level (aged 40–79)				
Mortality rate difference (RD; per 100,000 person-years)^a^	313	(−60–686)	593	(235–951)
Mortality rate ratio (RR)^b^	1.23	(0.91–1.55)	1.55	(1.14–1.96)
Slope Index of Inequality (SII; per 100,000 person-years)	404	(356–453)	721	(668–774)
Relative Index of Inequality (RII)	1.35	(1.30–1.39)	1.75	(1.68–1.82)
Occupational class (aged 40–64)				
Mortality RD (per 100,000 person-years)^c^	98	(−106–301)	204	(−26–433)
Mortality RR^d^	1.18	(0.81–1.56)	1.67	(0.90–2.44)
Average Inter-group Difference (AID absolute version)^*^	18.4		46.2	
Average Inter-group Difference (AID relative version) (%)^*^	3.1		9.9	
Women				
Educational level (aged 40–79)				
Mortality RD (per 100,000 person-years)^a^	66	(−192–325)	216	(42–389)
Mortality RR^b^	1.09	(0.71–1.46)	1.38	(0.99–1.77)
SII (per 100,000 person-years)	85	(52–119)	194	(157–232)
RII	1.12	(1.08–1.18)	1.31	(1.23–1.39)
Occupational class (aged 40–64)				
Mortality RD^c^ (%)	8	(−117–118)	−89	(−228–49)
Mortality RR^d^	1.00	(0.51–1.50)	0.65	(0.28–1.03)
AID absolute version^*^	16.3		15.9	
AID relative version (%)^*^	6.5		7.8	

CI, confidence interval.

^a^Mortality rate difference (RD) was calculated as RD = mortality rate_(low)_ − mortality rate_(high)_ with age-standardized.

^b^Mortality rate ratio (RR) was estimated as RR = mortality rate_(low)_/mortality rate_(high)_ with age-standardized.

^c^Mortality rate difference (RD) was calculated as RD = mortality rate_(manual workers)_ − mortality rate_(upper non-manual workers)_ with age-standardized.

^d^Mortality rate ratio (RR) was calculated as RR = mortality rate_(manual workers)_/mortality rate_(upper non-manual workers)_ with age-standardized.

^*^Regarding the Average Inter-group Difference (AID), we calculated the point estimates only.

For men, the RD (per 100,000 person-years) between manual workers and upper non-manual workers increased from 98 (95% CI, −106 to 301) in wave 1 to 204 (95% CI, −26 to 433) in wave 2, and the RR also increased from 1.18 (95% CI, 0.81–1.56) in wave 1 to 1.67 (95% CI, 0.90–2.44) in wave 2. For women, the RD (per 100,000 person-years) between manual workers and upper non-manual workers reversed from 8 (95% CI, −117 to 118) in wave 1 to −89 (95% CI, −228 to 49) in wave 2, and the RR changed from 1.00 (95% CI, 0.51–1.50) in wave 1 to 0.65 (95% CI, 0.28–1.03) in wave 2. AIDs also indicated that inequality increased by occupational class: AID (relative version: Gini coefficient-like measure) increased from 3.1% in wave 1 to 9.9% in wave 2 for men. AID (relative version) also increased from 6.5% in wave 1 to 7.8% in wave 2 for women.

## DISCUSSION

### Main findings

This study is a novel attempt to estimate exact national mortality rates both by educational level and occupational class using longitudinal national census data linked with death records, which was evaluated by comparing to the complete national mortality registry in Japan. Our findings showed clear mortality differences by socioeconomic status persisted in Japan. In addition, inequality measures indicated mortality inequalities increased between 2000–2005 and 2010–2015 for men. For women, changes in the inequality indices showed the opposite directions for educational level (inequalities increased) and occupational class (inequalities reversed). Although estimates calculated through deterministic-linkage methods should be interpreted with caution, the linked mortality data presented in this study may, nevertheless, represent new estimates for assessing mortality inequalities by socioeconomic status in Japan.

### Interpretations

Our estimates should be compared to a previous study that assessed the changes in educational inequalities in mortality in Japan between 2000 and 2010.^18^ The results showed that men and women aged 40–75 years with primary and junior high school graduation had about 15–25% and 10–20% higher all-cause mortality, respectively, than counterparts with junior college and university graduation in 2000.^18^ Their conclusions that relative mortality inequalities persisted between 2000 and 2010 were also comparable with our results, though their observation periods of death records were 6 months and the method of deterministic linkage (1:n matching) was somewhat different from ours (1:1 matching), in addition to the category of the educational attainment information.^18^ Our estimates also confirmed the presence of similar inequality patterns, albeit with smaller differences in magnitude, in Japan when compared to estimates reported for other high-income countries.^3^^–^^12^ Our longitudinal mortality database may facilitate between-country comparative research of education-based mortality inequalities, because the education-classification method used in our database affords easy comparisons with other high-income countries.^3^^,^^4^^,^^8^ However, the generalizability of the Japanese-census-linked mortality data should be carefully considered. Our mortality database may underrepresent individuals living in large cities, as discussed below, whereas the longitudinal mortality data from other high-income countries generally cover the entire population.^3^^–^^12^

The national census’ missing data regarding educational attainment (wave 2: 12.1% for men and 11.4% for women aged 40–79 years) is expected for any census-linked longitudinal mortality data developed in Japan. These missing data may distort the validity of inequality estimates. For example, all-cause ASMRs (per 100,000 person-years) for individuals with low and unknown educational levels were 1,670 and 1,682 for men, and 777 and 699 for women, respectively. Even if more people with a low education level do not report their educational attainment, high amounts of missing data do not cause mortality among people with a low education level in our database to be underestimated because the estimated mortality of unknown educational level was similar and lower for men and women, respectively.

For exact estimates of mortality by occupational class, further analysis in which correction factors are applied to each worker is required.^28^ This is because unemployed people’s last occupation is unknown, and workers in lower occupational classes have a higher likelihood of being unemployed.^15^^,^^28^ For each study period, male upper non-manual workers had higher cancer mortality rates than male lower non-manual workers; this pattern is similar to that shown in a previous study.^15^ However, we also found that male upper non-manual workers had lower mortality rates from cardiovascular disease and external causes than male lower non-manual workers, which differs from the previous study.^15^ In addition, the inequality indices by occupational class (AIDs) changed in the opposite directions for our results (inequalities increased) and a previous study (inequalities decreased) between 2000–2005 and 2010–2015.^15^ This discrepancy may be due to under-sampling of urban-based workers supposing that managers and professional workers in urban regions experience heavy burdens in severe work environments. However, there is no clear evidence to explain the variations in occupational mortality across regions. Furthermore, in contrast to the well-documented male mortality by occupation,^15^^–^^17^ few studies have focused on female workers in Japan. Our findings suggest new estimates for female workers. We identified higher mortality among female upper non-manual workers than for female manual workers from each broad cause-specific death. This finding is comparable to unique male mortality inequalities by occupational class in Japan, which was confirmed using a cross-sectional design.^15^ Further analysis is necessary to discuss the applicability of making comparisons using mortality calculated from cross-sectional mortality data (existing national statistics) and linked longitudinal mortality data.

Estimation of socioeconomic inequalities by age group is another challenge for better understanding health inequalities. eFigure 3 shows that mortality gradients by educational level were substantial across all age groups. This figure implies relative inequalities in mortality were more prominent among the younger generation for both sexes. Because some estimates were identified as irregular (eg, women aged 65–69 in wave 2) in addition to overestimations of mortality in the younger generations, the trend is still under discussion; however, our mortality database suggests that mortality-related socioeconomic inequalities did not increase with age in Japan. This finding may contribute to the understanding of interactions between health and age socioeconomic stratification.

### Limitations

There are four major limitations to using census-linked longitudinal mortality data. The first concerns the generated IDs. If all individuals retained their residence and marital status during the follow-up period, unique IDs would afford complete matches with deceased persons (although a risk of misreporting would remain). However, according to the October 2015 census, 10.2% of Japan residents had moved to another municipality since October 2010.^19^ This rate was highest among people aged 30–34 years (31.5%) and lowest among those aged 70–74 (2.9%; eTable 9).^19^ According to 2010 National Vital Statistics, 140,428 people (700,214 couples) married and 502,756 people (251,378 couples) divorced, representing approximately 1.5% of the Japanese population.^20^ Although it is difficult to determine the exact number of people widowed per year, this suggests that at least 1.5–2.0% of the Japanese population changed marital status in 2010, indicating that approximately 10% of the population changed their marital status between 2010 and 2015. Allowing for address and marital-status change (approximately 10%, respectively), we estimated that approximately 20% of individuals changed ID between October 2010 and October 2015. Thus, approximately 80% of the sample population matched perfectly when the person died during the period, while approximately 20% may have been mismatched. Mismatch was more likely among younger generations (ie, 30–39 years), who had a higher likelihood of address change and marriage.

This mismatch may have caused an overestimation of mortality in our database. Changing ID causes both overestimation (because people who did not have a unique ID at the census developed a unique ID when they changed marital status or municipality during follow-up) and underestimation (because people who had a unique ID at the census lost this when they changed marital status or municipality during follow-up); however, overestimation is likely to have been more prominent due to the much larger number of individuals with duplicated IDs at the census. As shown in Table 1 and Table 2, we confirmed overestimations of mortality among young males (eg, over-ascertainment of deaths for men aged 35–39 years; note that the tendency was reversed among women aged 30–39 years in wave 2); therefore, to avoid inaccuracies in the estimations, we excluded all persons aged 30–39 years from the estimated mortality by socioeconomic status. However, systematic overestimation may still cause underestimation of mortality inequalities, especially in relative terms.

Second, we covered all prefectures in Japan, but individuals living in highly populous municipalities (ie, municipalities in Tokyo, Kanagawa, and Osaka) were underestimated, even though we applied a large weight to those sample populations. For example, no men aged 50–54 living in Hyogo Prefecture (a large prefecture) who had high education levels or were lower non-manual workers (*n* = 25,235 in the 2010 census) were included in the sample population. Despite aiming for a nationally representative sample, these missing data may distort the sample population and the generalizability of the mortality estimates. Thus, this mortality database may include under-representation for individuals living in the capital region and prefectures with large populations. We confirmed that the results (the mortality of complete registry and sample weighted mortality) were correlated; however, variations were observed by prefecture (eTable 10 and eTable 11). It is difficult to determine whether we underestimated or overestimated socioeconomic inequalities in mortality due to this bias because there is no evidence about differences in health inequalities between urban cities and rural areas in Japan.

Third, across all age categories highly educated persons had a high probability of changing their address (eTable 12)^19^; this would cause loss of unique IDs and under-ascertainment of deaths during the follow-up period. Therefore, mortality of highly educated men and women may be underestimated, resulting in overestimation of mortality inequalities. Thus, mortality inequalities should be interpreted with caution.

Fourth, the follow-up period also needs to be discussed. While a shorter follow-up period would bring a more complete linkage and possibly less bias, given the large proportion of the population who change IDs over time. Initially, we had tried shorter follow-up periods (1 year or 3 years after the census) before we performed this study with a follow-up period of 5 years. The results of shorter follow-up periods (ie, 1 or 3 years) showed weighted mortality rates were more overestimated than those of the complete registry. As we discussed above, changing ID causes both overestimation and underestimation and we concluded overestimation was more likely to be prominent for shorter follow-up periods in the current data.

### Conclusions

As a result of systematic over-ascertainment of deaths for the certain causes of death and demographic factors, our deterministic-linkage-based estimates between the Japanese population census and mortality records should be interpreted with caution. In particular, mortality inequalities may be biased by both mechanisms causing overestimation and underestimation. However, the developed census-linked longitudinal mortality data nevertheless produce new estimates for assessing mortality inequalities by socioeconomic status for the Japanese population. In addition, our estimates can be triangulated with estimates obtained with other methods. Further study is necessary to develop better national longitudinal mortality databases and provide benchmarks for monitoring and reducing socioeconomic inequalities in health.

## SUPPLEMENTARY MATERIAL

eTable 1. Distributions of the population of unique ID and duplicated ID from the census in 2010 and from death record between 2010 and 2015

eTable 2. Weight calculation for the analysis of the national census in 2010

eTable 3. Definitions of educational level

eTable 4. Definitions of occupational class

eTable 5. Distribution of population and all-cause age-standardized mortality rate (per 100,000 person-years)^a^, for men during 2000–2005

eTable 6. Distribution of population and all-cause age-standardized mortality rate (per 100,000 person-years)^a^, for women during 2000–2005

eTable 7. Comparison of broad cause-specific age-standardized mortality rate (per 100,000 person-years) by population during 2010–2015^*^

eTable 8. All-cause and broad cause-specific age-standardized mortality rate (per 100,000 person-years) by socioeconomic status during 2000–2005

eTable 9. Percentages of changing their address to another municipality between 2010 and 2015, %

eTable 10. Number, percentage, and mortality of all population and weighted sample population by prefecture, men (40–79 years) during 2010–2015

eTable 11. Number, percentage, and mortality of all population and weighted sample population by prefecture, women (40–79 years) during 2010–2015

eTable 12. Percentages of changing their address to another municipality by educational level between 2005 and 2010, %

eFigure 1. Deterministic linkage procedure for linking the population census and vital statistics data for 2000–2005.

eFigure 2. Estimated crude mortality rates by age and educational level, during 2000–2005

eFigure 3. Estimated crude mortality rates by age and educational level, during 2010–2015
