# Supplementary material for: Estimation of Socioeconomic Inequalities in Mortality in Japan Using National Census-linked Longitudinal Mortality Data
Source: J Epidemiol. 2023 May 5;33(5):246–55. doi: 10.2188/jea.JE20210106 (PMC10043154; doi:10.2188/jea.JE20210106)
Supplement: Supplementary file 1 [file je-33-246-s001.pdf]

eTable 1. Distributions of the population of unique ID and duplicated ID from the census in 2010 and from death record between 2010 and 2015

	The number of duplicated ID	From census in 2010			From death record between 2010 and 2015		
		Population	%	Cumulative percentage	Deceased	%	Cumulative percentage
**Unique ID (sample population)***	**1**	**1,537,337**	**1.9**	**1.9**	**886,807**	**28.8**	**28.8**
Duplicated ID**	2	1,692,122	2.1	4.0	527,226	17.1	45.9
Duplicated ID	3	1,668,558	2.1	6.1	345,705	11.2	57.1
Duplicated ID	4	1,599,268	2.0	8.0	243,560	7.9	65.0
Duplicated ID	5	1,515,970	1.9	9.9	183,045	5.9	71.0
Duplicated ID	6	1,433,394	1.8	11.7	141,924	4.6	75.6
Duplicated ID	7	1,363,047	1.7	13.4	113,519	3.7	79.3
Duplicated ID	8	1,301,600	1.6	15.0	92,464	3.0	82.3
Duplicated ID	9	1,243,611	1.5	16.5	76,599	2.5	84.8
Duplicated ID***	10	1,198,700	1.5	18.0	62,540	2.0	86.8
Duplicated ID	11	1,145,408	1.4	19.4	52,833	1.7	88.5
Duplicated ID	12	1,113,012	1.4	20.8	45,036	1.5	90.0
Duplicated ID	13	1,073,605	1.3	22.1	38,779	1.3	91.2
Duplicated ID	14	1,047,074	1.3	23.4	33,012	1.1	92.3
Duplicated ID	15	1,019,250	1.3	24.6	28,920	0.9	93.2
Duplicated ID	16	1,000,144	1.2	25.9	23,920	0.8	94.0
Duplicated ID	17	977,993	1.2	27.1	21,454	0.7	94.7
Duplicated ID	18	949,266	1.2	28.3	19,260	0.6	95.3
Duplicated ID	19	930,259	1.2	29.4	16,511	0.5	95.9
Duplicated ID	20-49	20,597,175	25.4	54.4	124,481	4.0	99.9
Duplicated ID	50 and over	36,578,583	45.2	100.0	2,527	0.1	100.0
	(Total)	80,985,376	100,00	-	3,080,122	100	-

*This means there are 1,537,337 persons who have unique ID: 1,537,337 (=1,537,337/1) kinds of unique ID.

**This means there are 1,692,122 persons who have two-duplicated ID: 846,061 (=1,692,122/2) kinds of two-duplicated ID.

*** This means there are 1,198,700 persons who have ten-duplicated ID: 119,870 (=1,198,700/10) kinds of ten-duplicated ID.

eTable 2. Weight calculation for the analysis of the national census in 2010

Number (Weighting key)	Prefecture (n=47)	Sex (n=2)	Age category (n=10)	Marital status (n=5)	Educational level (n=4)	Occupational class (n=8), aged 30–64 years only	Number of population (A)	Number of sample (B)	Weight calculation (A/B)	*Allocated weight*
1	Hokkaido	Men	30–34	Widow	High	Manual worker	2	2	1	*1 (minimum)*
2	Yamaguchi	Men	30–34	Widow	Middle	Lower non-manual worker	3	3	1	*1*
.	.	.	.	.	.	.	.	.	.	.
25990	Kyoto	Women	30–34	Unknown	High	Unemployment	9	2	4.5	*5*
25991	Hyogo	Men	75–79	Unknown	High	—	18	4	4.5	*5*
.	.	.	.	.	.	.	.	.	.	.
47740	Kagawa	Men	45–49	Single	Low	Manual worker	199	10	19.9	*20*
47741	Saga	Women	65–69	Widow	Middle	—	2,488	125	19.9	*20*
.	.	.	.	.	.	.	.	.	.	.
60322	Miyazaki	Men	30–34	Married	High	Lower non-manual worker	2,699	27	99.9	*100*
60323	Hiroshima	Women	40–44	Divorced	Unknown	Upper non-manual worker	100	100	100	*100*
.	.	.	.	.	.	.	.	.	.	.
71858	Chiba	Men	60–64	Married	Low	Upper non-manual worker	9,845	1	9,845	*9,845*
71859	Saitama	Men	55–59	Married	High	Upper non-manual worker	10,086	1	10,086	*10,000*
.	.	.	.	.	.	.	.	.	.	.
71991	Kanagawa	Women	60–64	Married	High	Unemployment	43,090	1	43,090	*10,000 (maximum)*
.	.	.	.	.	.	.	.	.	.	.
97424	Saga	Women	40–44	Married	Low	Unemployment	172	0	—	—

* The weight range was set between 1 and 10,000; all weight above 10,000 was allocated as 10,000.

eTable 3. Definitions of educational level

Educational level	Educational background defined by the Japanese Population Census	Correspondence to the International Standard Classification of Education (ISCED), 2011
Low	Elementary school/Junior high school graduates	ISCED 1: Primary education<br>ISCED 2: Lower secondary education
Middle	High school graduates	ISCED 3: Upper secondary education
	Technical professional school graduates	ISCED 4: Post-secondary non-tertiary education
High	2-year college graduates	ISCED 5: Short-cycle tertiary education
	University graduates	ISCED 6: Bachelor’s or equivalent level
	Graduate school	ISCED 7: Master’s or equivalent level
		ISCED 8: Doctoral or equivalent level

eTable 4. Definitions of occupational class

Occupational class	Occupation defined by Japanese Standard Occupational Classification (JSOC)	Employment status	Correspondence to the Erikson-Goldthorpe-Portocarero (EGP) scheme
Upper non-manual workers	(A) Administrative and managerial workers<br>(B) Professional and engineering workers	-<br>-<br>-	I: Higher-grade professionals, administrators and officials; managers in large industrial establishments; large proprietors<br>II: Lower-grade professionals, administrators and officials; higher-grade technicians; managers in small business and industrial establishments; supervisors of non-manual employees
Lower non-manual workers	(C) Clerical workers<br>(D) Sales workers<br>(E) Service workers	-<br>-	III: Routine non-manual employees in administration and commerce; sales personnel; other rank-and-file service workers
Manual workers	(H) Manufacturing process workers<br>(I) Transport and machine operating workers<br>(J) Construction and mining workers<br>(K) Carrying, cleaning, packaging, and related workers	-<br>-<br>-<br>-	V/VI: Lower-grade technicians; supervisors of manual workers; skilled manual workers<br>VIIa: Semi- and unskilled manual workers (not in agriculture)
Farmers	(G) Agriculture forestry and fishery workers	-	IVc: Farmers and smallholders; self-employed fishermen<br>VIIb: Agricultural workers
Self-employed	-	Self-employed with employees, self-employed no employees	IVa: Small proprietors; artisans, etc., with employees<br>IVb: Small proprietors, artisans, etc., without employees

eTable 5. Distribution of population and all-cause age-standardized mortality rate (per 100,000 person-years)[a], for men during 2000–2005

	All population (complete registry)			Sample population					Comparisons with complete registry
				Unweighted			Weighted		
	Population	(%)	(A) ASMR	Number of sample	(%)	ASMR	(%)	(B) ASMR	% difference [(B)-(A)]/(A) (%)
Total (30–79 years)	37,472,213		-	1,238,087		-		-	
Total (40–79 years)	29,092,384		1,520	1,011,512		1,882		1,530	0.7
Age, years									
30–34	4,354,603	11.6	98	105,161	8.5	217	8.6	115	17.8
35–39	4,025,226	10.7	142	121,414	9.8	295	10.0	145	2.0
40–44	3,873,623	10.3	222	126,992	10.3	457	10.4	300	35.5
45–49	4,425,700	11.8	359	134,175	10.8	690	11.0	354	-1.5
50–54	5,174,238	13.8	568	137,473	11.1	1,059	11.2	614	8.1
55–59	4,264,397	11.4	874	131,312	10.6	1,406	10.7	1,062	21.6
60–64	3,731,102	10.0	1,296	127,069	10.3	1,903	10.3	1,367	5.5
65–69	3,344,958	8.9	2,142	123,010	9.9	2,676	9.9	1,897	-11.4
70–74	2,660,796	7.1	3,460	116,763	9.4	3,737	9.2	3,482	0.7
75–79	1,617,570	4.3	5,592	114,718	9.3	5,229	8.8	5,398	-3.5
Marital status (40–79 years)									
Single	2,431,173	8.4	3,258	264,013	26.1	2,211	9.0	2,307	-29.2
Married	24,152,549	83.0	1,324	179,702	17.8	1,368	81.7	1,410	6.6
Widow	962,426	3.3	2,496	248,619	24.6	1,894	3.5	1,986	-20.4
Divorced	1,117,629	3.8	3,753	239,352	23.7	2,405	4.1	2,670	-28.9
Unknown	428,607	1.5	120	79,826	7.9	80	1.7	75	-37.9
Educational level (40–79 years)[b]									
High (ISCED: 5–8)	7,197,959	24.7	-	102,695	10.2	1,835	3.0	1,097	
Middle (ISCED: 3, 4)	12,521,813	43.0	-	367,048	36.3	1,960	24.7	1,371	
Low (ISCED: 1, 2)	8,198,114	28.2	-	470,514	46.5	2,049	43.9	1,581	
Unknown	1,174,498	4.0	-	71,255	7.0	727	28.3	1,684	
Occupational class (EGP scheme, 40–64 years)[c]									
Upper non-manual workers (I+II)	2,899,781	13.9	-	40,701	6.3	745	13.9	535	
Lower non-manual workers (III)	5,228,540	25.1	-	82,437	12.8	906	25.3	594	
Manual workers (V+VI+VIIa)	6,433,912	30.9	-	225,435	35.0	925	30.9	633	
Farmers (IVc+VIIb)	736,431	3.5	-	53,998	8.4	848	3.6	606	
Self-employed (Iva+b)	2,674,785	12.8	-	63,091	9.8	1,019	12.7	575	
Unemployment	2,856,742	13.7	-	178,738	27.7	1,453	13.6	1,512	

ASMR, age-standardized mortality rate.

[a] ASMR (per 100,000 person-years) was calculated from death between October 2000 and September 2005 counted by the vital statistics. Age-standardized mortality rates were computed using the 2013 European standard population and data in 5-

[b] Defined by the International Standard Classification of Education (ISCED)

[c] Defined by the Erikson-Goldthorpe-Portocarero (EGP) scheme

eTable 6. Distribution of population and all-cause age-standardized mortality rate (per 100,000 person-years)[a], for women during 2000–2005

	All population (complete registry)			Sample population					Comparisons with complete registry
				Unweighted			Weighted		
	Population	(%)	(A) ASMR	Number of sample	(%)	ASMR	(%)	(B) ASMR	% difference [(B)-(A)]/(A) (%)
Total (30–79 years)	39,450,864		-	1,310,058		-		-	
Total (40–79 years)	31,275,308		702	1,074,911		803		751	6.9
Age, years									
30–34	4,237,697	10.7	51	113,409	8.7	98	5.7	60	18.0
35–39	3,937,859	10.0	73	121,738	9.3	133	6.7	84	14.6
40–44	3,818,683	9.7	111	124,644	9.5	190	7.6	97	-13.0
45–49	4,404,042	11.2	176	132,017	10.1	284	8.6	248	40.3
50–54	5,198,029	13.2	262	136,899	10.4	397	9.5	320	22.4
55–59	4,418,262	11.2	365	136,020	10.4	502	10.5	404	10.5
60–64	3,966,700	10.1	549	135,924	10.4	720	11.4	602	9.7
65–69	3,734,428	9.5	904	134,265	10.2	1,096	12.4	896	-0.9
70–74	3,219,578	8.2	1,545	134,448	10.3	1,670	13.3	1,577	2.1
75–79	2,515,586	6.4	2,877	140,694	10.7	2,619	14.3	3,081	7.1
Marital status (40–79 years)									
Single	1,573,015	5.0	1,436	248,180	23.1	1,183	5.4	1,222	-14.9
Married	23,035,189	73.7	540	189,355	17.6	565	72.2	599	10.9
Widow	4,595,493	14.7	920	302,991	28.2	850	15.2	953	3.5
Divorced	1,783,036	5.7	1,153	253,838	23.6	875	6.1	1,004	-12.9
Unknown	288,575	0.9	69	80,547	7.5	42	1.0	36	-48.5
Educational level (40–79 years)[b]									
High (ISCED: 5–8)	4,938,353	15.8	-	103,537	9.6	752	15.3	749	
Middle (ISCED: 3, 4)	15,326,318	49.0	-	414,518	38.6	785	50.0	743	
Low (ISCED: 1, 2)	9,855,126	31.5	-	501,995	46.7	865	32.0	815	
Unknown	1,155,511	3.7	-	54,861	5.1	555	2.7	852	
Occupational class (EGP scheme, 40–64 years)[c]									
Upper non-manual workers (I+II)	1,475,311	6.8	-	47,573	7.2	284	6.6	217	
Lower non-manual workers (III)	6,357,006	29.3	-	185,409	28.0	303	30.0	286	
Manual workers (V+VI+VIIa)	3,286,035	15.2	-	119,200	18.0	295	15.0	217	
Farmers (IVc+VIIb)	667,827	3.1	-	31,288	4.7	260	3.1	264	
Self-employed (IVa+b)	931,900	4.3	-	47,042	7.1	371	4.1	261	
Unemployment	8,948,883	41.3	-	232,565	35.1	602	41.2	429	

ASMR, age-standardized mortality rate.

[a] ASMR (per 100,000 person-years) was calculated from death between October 2000 and September 2005 counted by the vital statistics. Age-standardized mortality rates were computed using the 2013 European standard population and data in 5-

[b] Defined by the International Standard Classification of Education (ISCED)

[c] Defined by the Erikson-Goldthorpe-Portocarero (EGP) scheme

eTable 7. Comparison of broad cause-specific age-standardized mortality rate (per 100,000 person-years) by population during 2010–2015*

	All population (complete registry)	Weighted sample population	Comparisons with complete registry	
			Absolute difference	% difference [(B)-(A)]/(A) (%)
Men (40–79 years)				
All-cause	1,520	1,530	10	0.7
Broad cause death				
Cancer	620	583	-37	-6.0
Cardiovascular disease	401	413	12	3.1
External causes	118	137	19	16.4
Others	382	395	13	3.5
Women (40–79 years)				
All-cause	702	751	48	6.9
Broad cause death				
Cancer	281	271	-10	-3.4
Cardiovascular disease	206	225	19	9.2
External causes	45	51	6	14.2
Others	172	202	31	17.7

* Age-standardized mortality rates were computed using the 2013 European standard population and data in 5-year age intervals.

eTable 8. All-cause and broad cause-specific age-standardized mortality rate (per 100,000 person-years) by socioeconomic status during 2000–2005

	All-cause	95% CI	Cancer	95% CI	Cardiovascular disease	95% CI	External causes	95% CI	Others	95% CI
Men										
Total (40–79 years)	1,530	(1,517 - 1,542)	583	(575 - 591)	413	(406 - 419)	137	(133 - 141)	395	(389 - 402)
Total (40–64 years)	716	(707 - 725)	279	(273 - 284)	169	(165 - 174)	110	(107 - 114)	157	(152 - 161)
Educational level (40–79 years)[b]										
High (ISCED: 5–8)	1,371	(1,341 - 1,400)	564	(545 - 583)	345	(329 - 360)	93	(85 - 100)	367	(351 - 383)
Middle (ISCED: 3, 4)	1,581	(1,560 - 1,603)	606	(593 - 619)	448	(437 - 459)	134	(128 - 139)	392	(381 - 403)
Low (ISCED: 1, 2)	1,684	(1,662 - 1,706)	619	(606 - 632)	442	(431 - 454)	186	(177 - 194)	435	(424 - 447)
Unknown	1,097	(1,041 - 1,153)	241	(215 - 267)	249	(223 - 276)	117	(98 - 136)	486	(448 - 524)
Occupational class (EGP scheme, 40–64 years)[c]										
Upper non-manual workers (I+II)	535	(512 - 559)	280	(263 - 297)	106	(94 - 117)	69	(60 - 78)	78	(70 - 86)
Lower non-manual workers (III)	594	(574 - 614)	241	(228 - 255)	122	(113 - 130)	101	(93 - 108)	131	(121 - 140)
Manual workers (V+VI+VIIa)	633	(616 - 650)	262	(251 - 273)	166	(158 - 175)	114	(107 - 121)	90	(83 - 96)
Farmers (IVc+VIIb)	606	(560 - 653)	224	(197 - 250)	101	(83 - 120)	171	(145 - 197)	99	(80 - 117)
Self-employed (IVa+b)	575	(552 - 599)	219	(205 - 234)	158	(146 - 170)	79	(70 - 87)	118	(108 - 129)
Unemployment	1,512	(1,470 - 1,554)	425	(404 - 446)	378	(357 - 399)	238	(219 - 256)	470	(446 - 493)

eTable 8. Continued

	All-cause	95% CI			Cancer	95% CI			Cardiovascular disease	95% CI			External causes	95% CI			Others	95% CI		
Women																				
Total (40–79 years)	751	(743	-	758)	271	(266	-	276)	225	(220	-	229)	51	(49	-	53)	202	(198	-	206)
Total (40–64 years)	325	(319	-	331)	141	(137	-	145)	71	(68	-	74)	35	(33	-	37)	76	(73	-	79)
Educational level (40–79 years)[b]																				
High (ISCED: 5–8)	749	(717	-	780)	259	(240	-	278)	205	(187	-	222)	47	(39	-	54)	233	(215	-	250)
Middle (ISCED: 3, 4)	743	(731	-	755)	281	(273	-	288)	214	(207	-	220)	47	(44	-	50)	201	(195	-	207)
Low (ISCED: 1, 2)	815	(800	-	829)	271	(263	-	279)	259	(251	-	267)	55	(51	-	59)	228	(221	-	236)
Unknown	852	(806	-	899)	264	(239	-	290)	300	(272	-	327)	60	(47	-	72)	221	(196	-	246)
Occupational class (EGP scheme, 40–64 years)[c]																				
Upper non-manual workers (I+II)	217	(187	-	246)	89	(73	-	106)	46	(30	-	62)	21	(13	-	29)	52	(36	-	69)
Lower non-manual workers (III)	286	(273	-	298)	156	(147	-	165)	65	(59	-	71)	28	(25	-	31)	37	(33	-	41)
Manual workers (V+VI+VIIa)	217	(204	-	231)	101	(92	-	111)	45	(39	-	52)	38	(32	-	43)	30	(25	-	35)
Farmers (IVc+VIIb)	264	(232	-	295)	136	(114	-	158)	22	(14	-	29)	87	(67	-	107)	12	(6	-	18)
Self-employed (IVa+b)	261	(235	-	288)	123	(105	-	141)	59	(47	-	72)	19	(11	-	26)	49	(38	-	61)
Unemployment	429	(418	-	440)	159	(152	-	166)	105	(99	-	111)	37	(34	-	41)	128	(122	-	134)

ASMR, age-standardized mortality rate; CI, confidence interval.

[a] ASMR (per 100,000 person-years) was calculated from death between October 2000 and September 2005 counted by the vital statistics. Age-standardized mortality rates were computed using the 2013

[b] Defined by the International Standard Classification of Education (ISCED)

[c] Defined by the Erikson-Goldthorpe-Portocarero (EGP) scheme

eTable 9. Percentages of changing their address to another municipality between 2010 and 2015, %

Age, years	Total	Men	Women
30–34	31.5	30.9	32.2
35–39	21.9	22.3	21.5
40–44	13.8	15.0	12.6
45–49	9.8	11.5	8.1
50–54	7.7	9.4	6.1
55–59	6.2	7.4	4.9
60–64	4.8	5.6	4.1
65–69	3.6	4.0	3.3
70–74	2.9	3.0	2.9
75–79	3.0	2.7	3.2
Total (30–79)	10.2	11.1	9.4

*Data from the Population Census

eTable 10. Number, percentage, and mortality of all population and weighted sample population by prefecture, men (40–79 years) during 2010–2015

Prefecture		All population (complete registry)			Sample population			Comparisons with complete registry
						Weighted		
Number	Name	Population*	(%)	(A) ASMR	Number of sample	(%)	(B) ASMR	% difference [(B)-(A)]/(A) (%)
	Total	30,764,583		1,289	641,173		1,373	6.5
1	Hokkaido	1,356,334	4.4	1,323	60,954	5.0	1,255	-5.1
2	Aomori	344,484	1.1	1,581	13,712	1.2	1,690	6.9
3	Iwate	334,647	1.1	1,480	11,551	1.1	1,452	-1.8
4	Miyagi	556,079	1.8	1,369	13,757	1.9	1,404	2.6
5	Akita	281,900	0.9	1,398	8,078	1.0	1,422	1.7
6	Yamagata	293,948	1.0	1,259	11,509	1.0	1,402	11.4
7	Fukushima	500,988	1.6	1,381	19,685	1.8	1,111	-19.6
8	Ibaraki	744,341	2.4	1,328	15,421	1.9	1,140	-14.2
9	Tochigi	497,248	1.6	1,360	9,220	1.0	1,023	-24.8
10	Gunma	494,763	1.6	1,315	11,680	1.8	1,155	-12.1
11	Saitama	1,796,420	5.8	1,280	26,497	5.7	1,614	26.0
12	Chiba	1,532,187	5.0	1,252	20,601	5.2	1,215	-2.9
13	Tokyo	3,043,712	9.9	1,284	16,103	10.3	1,424	10.9
14	Kanagawa	2,200,995	7.2	1,225	18,380	7.0	1,533	25.1
15	Niigata	595,734	1.9	1,263	12,307	2.0	1,266	0.2
16	Toyama	269,487	0.9	1,275	5,136	0.9	933	-26.8
17	Ishikawa	276,223	0.9	1,240	6,478	0.8	1,097	-11.5
18	Fukui	193,061	0.6	1,198	5,738	0.7	966	-19.4
19	Yamanashi	212,557	0.7	1,250	8,894	0.8	1,187	-5.1
20	Nagano	528,478	1.7	1,108	23,597	1.9	962	-13.2
21	Gifu	502,462	1.6	1,214	14,431	1.8	1,192	-1.8
22	Shizuoka	937,136	3.0	1,243	14,801	3.0	1,808	45.5
23	Aichi	1,734,501	5.6	1,252	24,613	5.7	1,354	8.2
24	Mie	448,789	1.5	1,254	10,350	1.5	933	-25.6
25	Shiga	321,620	1.0	1,144	6,998	1.0	1,316	15.1
26	Kyoto	603,381	2.0	1,234	12,954	2.1	1,115	-9.7
27	Osaka	2,087,621	6.8	1,396	23,881	5.9	1,963	40.6
28	Hyogo	1,321,417	4.3	1,274	16,913	2.6	2,232	75.2
29	Nara	337,883	1.1	1,182	12,893	1.2	1,065	-9.8
30	Wakayama	245,874	0.8	1,378	10,394	0.9	1,088	-21.0
31	Tottori	141,234	0.5	1,360	6,372	0.5	1,048	-22.9
32	Shimane	178,454	0.6	1,268	6,669	0.6	1,287	1.5
33	Okayama	456,311	1.5	1,242	9,886	1.6	957	-23.0
34	Hiroshima	675,193	2.2	1,247	10,680	2.1	655	-47.5

35	Yamaguchi	354,835	1.2	1,330	6,440	1.2	1,503	13.0
36	Tokushima	194,413	0.6	1,334	8,962	0.7	1,128	-15.4
37	Kagawa	240,014	0.8	1,247	6,166	0.8	941	-24.6
38	Ehime	346,110	1.1	1,345	6,996	1.1	951	-29.3
39	Kochi	187,231	0.6	1,358	12,053	0.7	1,104	-18.7
40	Fukuoka	1,149,180	3.7	1,310	26,097	4.1	1,374	4.9
41	Saga	198,349	0.6	1,322	7,063	0.6	1,202	-9.0
42	Nagasaki	345,586	1.1	1,322	7,170	1.1	1,068	-19.2
43	Kumamoto	426,422	1.4	1,198	15,187	1.5	1,217	1.5
44	Oita	286,313	0.9	1,232	6,124	0.9	1,040	-15.6
45	Miyazaki	273,470	0.9	1,282	8,838	0.9	1,456	13.5
46	Kagoshima	411,318	1.3	1,348	14,599	1.5	1,360	0.9
47	Okinawa	305,880	1.0	1,333	14,345	1.1	1,235	-7.4

ASMR, age-standardized mortality rate.

* Number of populations in 2010 by the Population Census.

** ASMR (per 100,000 person-years) was calculated from death between October 2010 and September 2015 counted by the vital statistics. ASMRs were computed using the 2013 European standard population and data in 5-year age intervals.

eTable 11. Number, percentage, and mortality of all population and weighted sample population by prefecture, women (40–79 years) during 2010–2015

Prefecture		All population (complete registry)			Sample population			Comparisons with complete registry
						Weighted		
Number	Name	Population*	(%)	(A) ASMR	Number of sample	(%)	(B) ASMR	% difference [(B)-(A)]/(A) (%)
	Total	32,575,276		595	637,817		632	6.2
1	Hokkaido	1,546,617	4.7	613	63,924	5.3	606	-1.1
2	Aomori	394,863	1.2	657	13,582	1.3	558	-15.1
3	Iwate	366,532	1.1	694	11,665	1.2	814	17.3
4	Miyagi	593,185	1.8	689	13,588	1.9	694	0.7
5	Akita	318,126	1.0	593	8,151	1.1	490	-17.3
6	Yamagata	311,904	1.0	559	11,486	1.0	494	-11.6
7	Fukushima	526,910	1.6	631	19,778	1.8	521	-17.5
8	Ibaraki	743,543	2.3	627	16,108	1.7	758	20.8
9	Tochigi	499,128	1.5	640	9,280	0.9	621	-3.0
10	Gunma	507,095	1.6	607	11,813	1.7	578	-4.7
11	Saitama	1,783,971	5.5	623	25,711	5.3	765	22.9
12	Chiba	1,538,775	4.7	600	20,369	5.0	609	1.5
13	Tokyo	3,118,302	9.6	590	12,794	10.2	716	21.5
14	Kanagawa	2,192,643	6.7	582	17,040	6.2	632	8.6
15	Niigata	625,846	1.9	541	12,731	2.0	540	-0.2
16	Toyama	292,192	0.9	549	5,242	0.9	363	-33.9
17	Ishikawa	300,792	0.9	566	6,501	0.8	632	11.8
18	Fukui	206,345	0.6	537	5,694	0.7	394	-26.6
19	Yamanashi	219,766	0.7	576	9,718	0.7	509	-11.6
20	Nagano	551,280	1.7	518	24,497	1.9	494	-4.6
21	Gifu	538,850	1.7	588	14,608	1.8	602	2.4
22	Shizuoka	966,182	3.0	580	14,415	3.0	485	-16.3
23	Aichi	1,751,763	5.4	603	23,942	5.3	545	-9.7
24	Mie	476,647	1.5	576	10,488	1.5	385	-33.1
25	Shiga	333,723	1.0	543	6,867	1.0	533	-1.7
26	Kyoto	662,850	2.0	579	12,175	2.2	702	21.4
27	Osaka	2,244,505	6.9	635	20,760	6.5	701	10.5
28	Hyogo	1,447,981	4.4	593	15,612	2.7	654	10.2
29	Nara	378,046	1.2	574	13,242	1.3	612	6.5
30	Wakayama	278,693	0.9	636	10,796	0.9	593	-6.9
31	Tottori	154,175	0.5	553	6,442	0.5	407	-26.4
32	Shimane	190,466	0.6	530	6,873	0.6	493	-6.9
33	Okayama	494,579	1.5	544	10,016	1.6	444	-18.4
34	Hiroshima	727,494	2.2	564	10,302	2.2	583	3.3

35	Yamaguchi	402,443	1.2	591	5,989	1.3	752	27.2
36	Tokushima	212,805	0.7	599	9,002	0.7	508	-15.2
37	Kagawa	260,479	0.8	571	6,622	0.8	401	-29.8
38	Ehime	392,187	1.2	600	6,788	1.2	708	18.1
39	Kochi	210,428	0.6	580	12,561	0.7	585	0.8
40	Fukuoka	1,310,760	4.0	598	25,024	4.3	756	26.4
41	Saga	223,757	0.7	591	7,568	0.7	415	-29.9
42	Nagasaki	393,906	1.2	588	7,512	1.2	492	-16.2
43	Kumamoto	483,575	1.5	541	15,947	1.6	545	0.8
44	Oita	324,592	1.0	552	6,191	1.0	440	-20.3
45	Miyazaki	308,162	0.9	567	8,938	1.0	520	-8.4
46	Kagoshima	456,217	1.4	609	15,114	1.5	711	16.9
47	Okinawa	312,196	1.0	582	14,351	1.1	512	-12.1

ASMR, age-standardized mortality rate.

* Number of populations in 2010 by the Population Census.

** ASMR (per 100,000 person-years) was calculated from death between October 2010 and September 2015 counted by the vital statistics. ASMRs were computed using the 2013 European standard population and data in 5-year age intervals.

eTable 12. Percentages of changing their address to another municipality by educational level between 2005 and 2010, %

Age, years	Men			Women		
	High (ISCED: 5–8)	Middle (ISCED: 3, 4)	Low (ISCED: 1, 2)	High (ISCED: 5–8)	Middle (ISCED: 3, 4)	Low (ISCED: 1, 2)
30–34	33.6	18.5	16.6	32.4	21.0	17.5
35–39	25.2	14.2	13.0	21.9	13.7	12.1
40–44	18.7	10.1	10.3	13.1	8.3	8.7
45–49	13.0	7.9	8.4	7.2	5.3	6.3
50–54	10.0	6.4	6.3	5.2	4.2	4.6
55–59	8.0	5.2	4.6	4.8	3.7	3.3
60–64	6.7	4.3	3.7	4.6	3.3	2.8
65–69	4.4	3.0	2.8	3.7	2.7	2.3
70–74	3.2	2.3	2.1	3.3	2.5	2.1
75–79	2.9	2.2	1.9	3.8	2.8	2.4
Total (30–79)	15.8	7.6	4.6	14.3	6.1	3.3

*Data from the Population Census

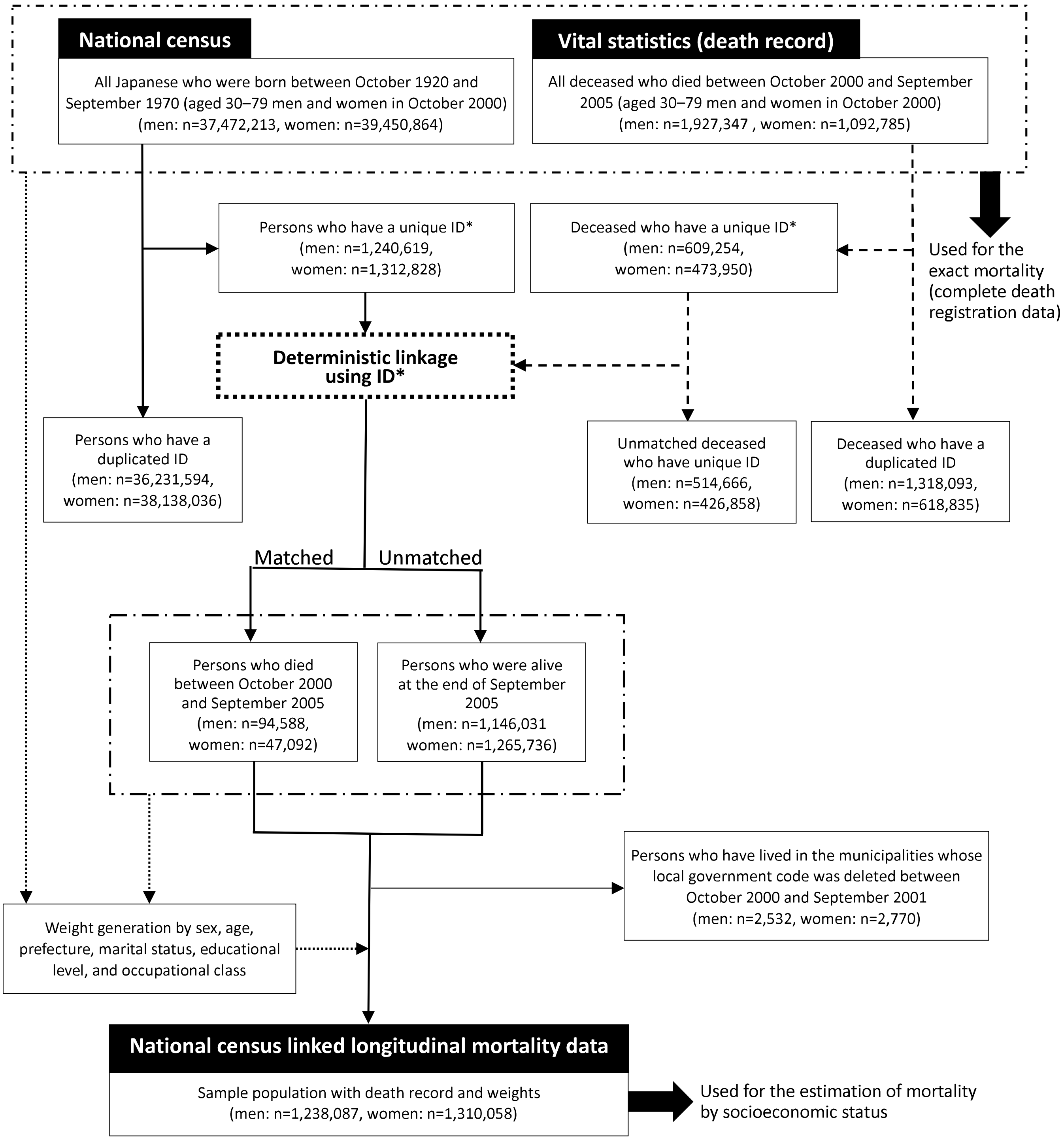

*ID (personal identification) was defined by sex, birth year, birth month, local government code, and marital status

eFigure 1. Deterministic linkage procedure for linking the population census and vital statistics data for 2000–2005.

(A) Men

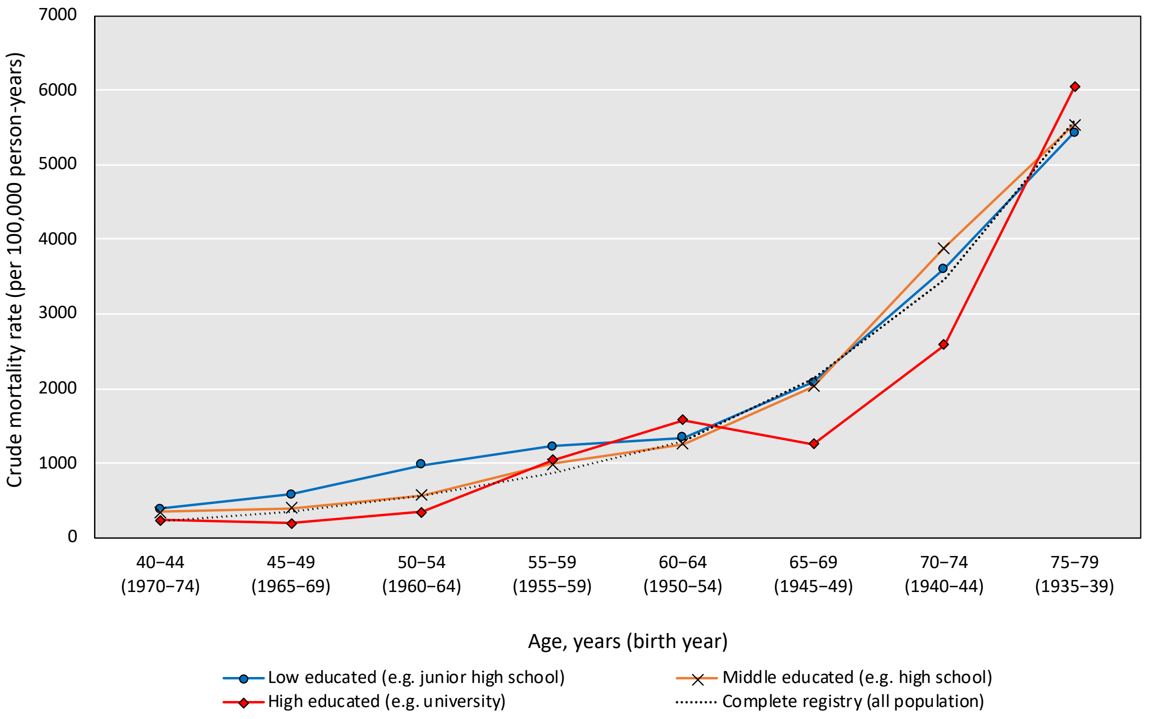

(B) Women

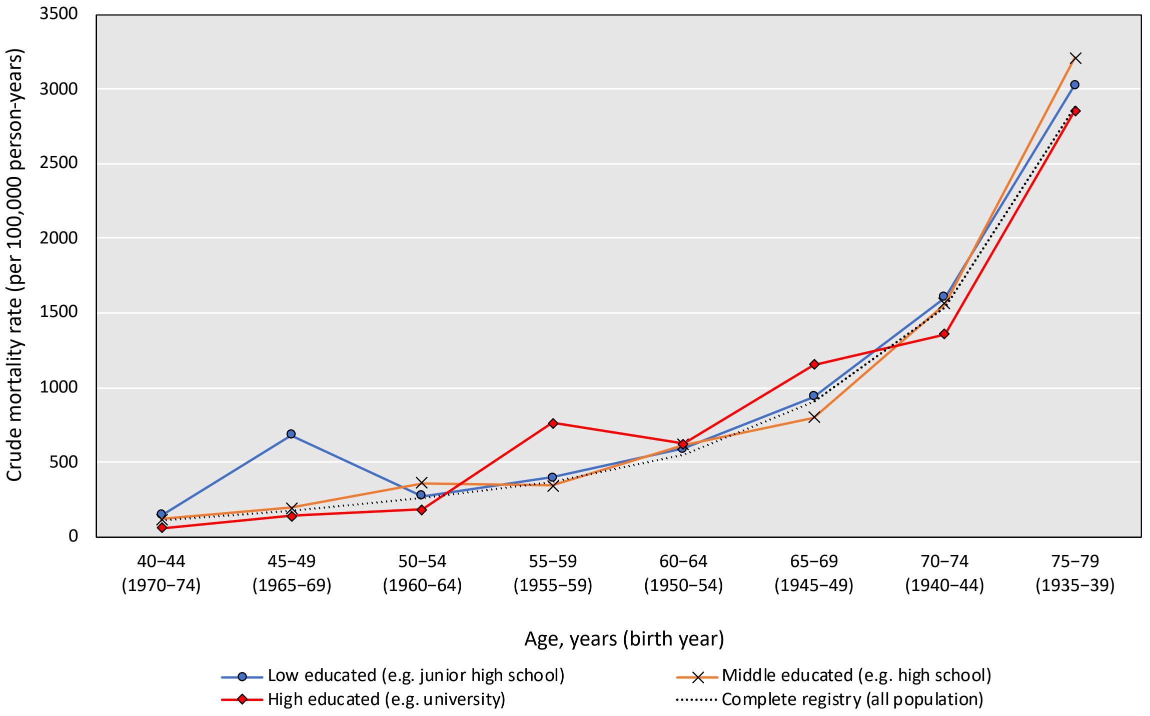

eFigure 2. Estimated crude mortality rates by age and educational level, during 2000–2005

(A) Men

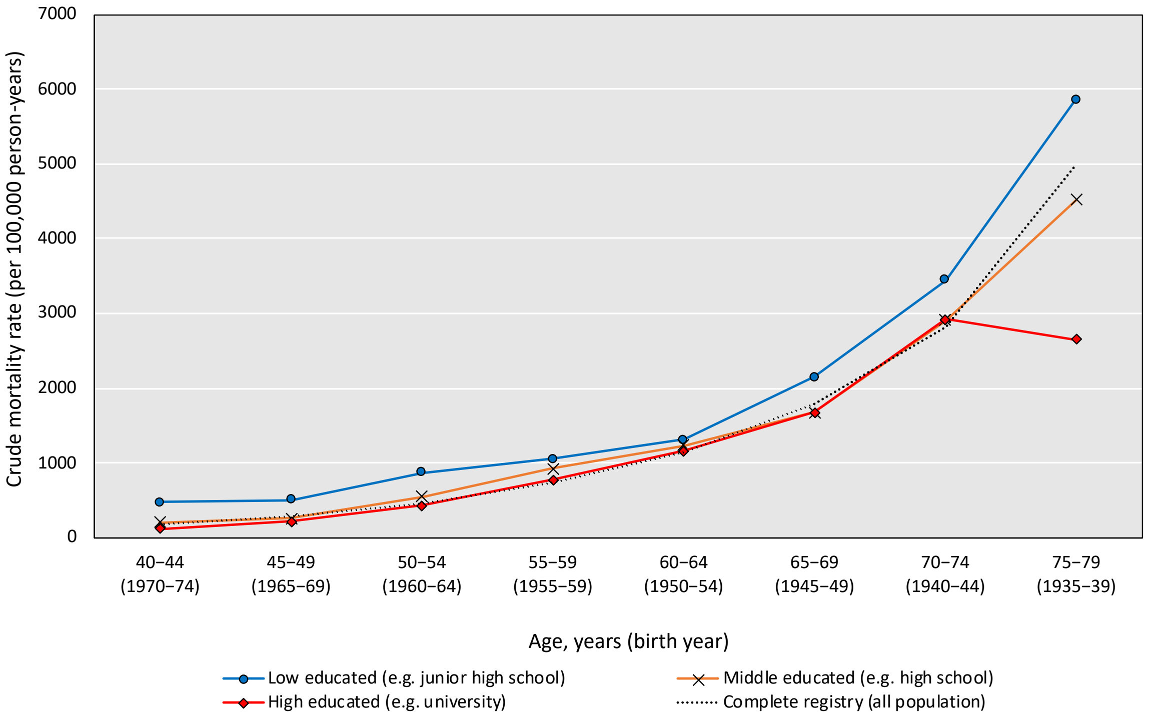

(B) Women

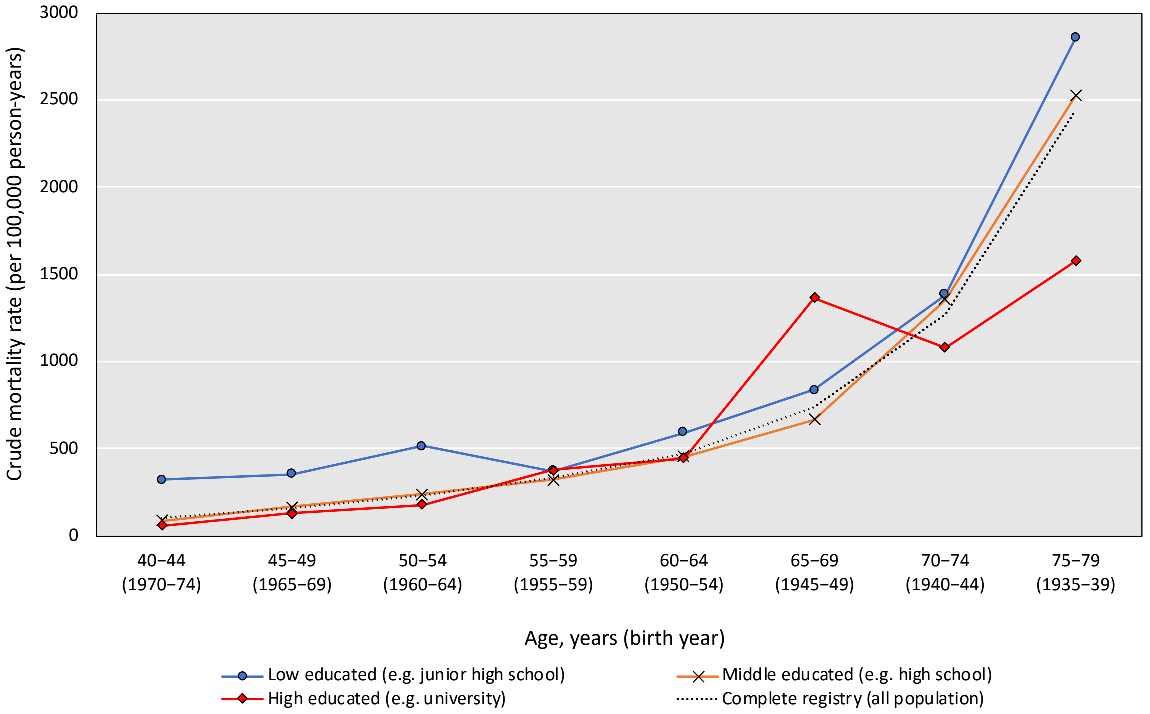

eFigure 3. Estimated crude mortality rates by age and educational level, during 2010–2015
